# The immune mechanism of the nasal epithelium in COVID-19–related olfactory dysfunction

**DOI:** 10.3389/fimmu.2023.1045009

**Published:** 2023-07-17

**Authors:** Shunmei Chen, Shufen Wang

**Affiliations:** Biomedical Engineering Research Institute, Kunming Medical University, Kunming, Yunnan, China

**Keywords:** COVID-19, olfactory dysfunction, SARS-CoV-2, nasal epithelium, immune mechanisms

## Abstract

During the first waves of the coronavirus disease 2019 (COVID-19) pandemic caused by severe acute respiratory syndrome coronavirus 2 (SARS-CoV-2) infection, olfactory dysfunction (OD) was reported as a frequent clinical sign. The nasal epithelium is one of the front-line protections against viral infections, and the immune responses of the nasal mucosa may be associated with OD. Two mechanisms underlying OD occurrence in COVID-19 have been proposed: the infection of sustentacular cells and the inflammatory reaction of the nasal epithelium. The former triggers OD and the latter likely prolongs OD. These two alternative mechanisms may act in parallel; the infection of sustentacular cells is more important for OD occurrence because sustentacular cells are more likely to be the entry point of SARS-CoV-2 than olfactory neurons and more susceptible to early injury. Furthermore, sustentacular cells abundantly express transmembrane protease, serine 2 (TMPRSS2) and play a major role in the olfactory epithelium. OD occurrence in COVID-19 has revealed crucial roles of sustentacular cells. This review aims to elucidate how immune responses of the nasal epithelium contribute to COVID-19–related OD. Understanding the underlying immune mechanisms of the nasal epithelium in OD may aid in the development of improved medical treatments for COVID-19–related OD.

## Introduction

Severe acute respiratory syndrome coronavirus 2 (SARS-CoV-2) is the coronavirus strain that caused the COVID-19 outbreak. According to clinical studies, the most prevalent COVID-19 symptoms consist of sore throat, fever, dyspnea, rhinorrhea, myalgia, headache, arthralgia, cough, shortness of breath, and diarrhea (1, 2). Loss of smell and taste was added to the list of COVID-19 disease symptoms by the WHO on May 4, 2020 (3). Olfactory dysfunction (OD) may occur before, during, or after the occurrence of common symptoms, with the occurrence of fever being associated with OD (2). During the first pandemic waves, the percentage of patients with OD or gustatory disorders (GD) as their only symptom in Germany, France, and China was 4%, 9%, and 12%, respectively (4). The percentage of patients presenting with OD or GD as their first symptom of infection in Germany, France, and China was 15%, 15%, and 23%, respectively (4). In addition, 25% of children had only OD or GD symptoms at COVID-19 presentation (4).

Meta-analysis studies observed that the prevalence of OD was greater than 47% in the acute phase of the infection during the first pandemic waves (5–8). OD appears to be a prevalent symptom of COVID-19. However, the genome of SARS-CoV-2 has been evolving for more than two years. The Omicron variant emerged in South Africa in 2021 and spread rapidly as the dominant global variant. The Omicron variant enhanced transmissibility and immune escape compared to the delta variant (9). One striking difference between the Omicron variant and previous variants was that the symptoms of OD were significantly less prevalent during the Omicron period (10). The low prevalence of OD during the Omicron wave, ranging from 5.8% to 16.7% according to sources (10–13), is worth paying attention to.

The objective of this review is to elucidate how immune mechanisms of the nasal epithelium contribute to COVID-19–related OD. This review will cover the infection and death of support cells in the olfactory epithelium because of their central role in the development of OD, but other plausible mechanisms of OD that are unrelated to immune responses, such as cilia dysfunction (14, 15), the loss of Bowman gland cells (16), the loss of energy supply (17), and the reduction of mucus (18, 19), are not covered in this review.

## General organization of the olfactory system

The olfactory system, one of the oldest principal sensory systems, is made up of the olfactory epithelium (OE), olfactory bulb, and olfactory cortex (20). The three functions of the olfactory system are sense of smell, immune function, and olfactory nerve regeneration (21). Odorant sensing is initiated in the OE, which comprises sensory neurons, sustentacular (SUS) cells, microvillar cells, and basal cells (20). The OE is one of the few sites containing neural stem cells in the adult nervous system (22). Olfactory sensory neurons (OSNs) are the primary sensing cells (23) located within the OE, and they are responsible for odorant detection. The axons of the OSNs project into the glomerular structures of the olfactory bulb, where they synapse with the dendrites of second-order neurons that in turn project their axons into the olfactory cortex (24), resulting in odorant perception, behaviors, and emotions (25). The major regions of the olfactory cortex are the anterior olfactory cortex, olfactory tubercle, entorhinal cortex, and piriform cortex (26). It is worth noting that epithelial cells express several pathogen-recognition receptors that trigger antiviral responses (24).

## Target cells of SARS-CoV-2 in the nasal epithelium

The genome of SARS-CoV-2 consists of 12 genes that encode 26 proteins (27). The viral genome is packaged into capsids capable of interfering with the host’s immune system function (28). The angiotensin-converting enzyme 2 (ACE2) has been identified as a receptor for SARS-CoV-2 to enter host cells, and it plays a crucial role in the pathogenesis of COVID-19 (29). ACE2 mediates virus attachment to target cells (30).

ACE2 is highly expressed in the nasal epithelium (31), which consists of the respiratory epithelium (RE) and OE. The former is thought to have a role in humidifying air as it enters the nasal cavity, and the latter is utilized for odor detection. Odor information is then relayed from the OE to the brain *via* OSN axons (32). Humans have approximately 10 million support cells and 10 million olfactory neurons in the OE (19). Immunostaining confirmed the results of single-cell sequencing that ACE2 is expressed in support cells, perivascular cells, and stem cells, rather than in neurons (32). ACE2 immunoreactivity has been found mainly on the SUS cells in both human and mouse OE, especially at the microvilli, apical surface, and supranuclear part of SUS cells (33). By confocal microscopy, Chen et al. demonstrated that most of ACE2 staining was localized in the OE, and co-staining markers for with immature and mature olfactory neurons demonstrated that ACE2 was not found in olfactory neurons (34). SUS cells are more likely to be the entry point of SARS-CoV-2 than olfactory neurons (35). Most studies show that SARS-CoV-2 virus mainly accumulates in SUS cells (36). SUS cells express high levels of ACE2 and transmembrane protease, serine 2 (TMPRSS2), which are the primary target of SARS-CoV-2 (34, 37). However, the SARS-CoV-2 virus has been evolving for a long time, and the variants of concern (VOCs) have been generated, including the Alpha (B.1.1.7; first VOC reported in the United Kingdom in late December 2020), Beta (B.1.351; first reported in South Africa in December 2020), Gamma (P.1; first reported in Brazil in early January 2021), Delta (B.1.617.2; first reported in India in December 2020), and Omicron (B.1.1.529; first reported in South Africa in November 2021) (38). VOCs have a better ability to survive and evade host defense mechanisms than the original virus (39). The most striking difference between the Omicron variant and previous variants was that the symptoms of OD were significantly less prevalent during Omicron’s prevalence.

An odorant-metabolizing enzyme—a glycosyltransferase encoded by the UGT2A1/A2 locus—is considered to be the host factor based on a recent genome-wide association study and meta-analysis (40, 41). A genome-wide association study (GWAS) identified that the *UGT2A1* and *UGT2A2* genes play a role in metabolizing odorants linked to OD (41). UGT2A1 and UGT2A2 are enzymes that belong to the uridine diphosphate glycosyltransferase family and metabolize lipophilic substrates (41). These enzymes are expressed in the OE and metabolize odorants that have bound to odorant receptors and may prevent saturation of these receptors (41). The SUS support cells expressed within the epithelium play a potential protective role against hazardous chemicals (42). UGT2A1 might be involved in the olfactory signal termination; the more it is metabolized, the less signal intensity is recorded (43). *UGT2A2* is a splice variant of *UGT2A1*, with identical c-termini and different N termini (41, 44). Polymorphisms in the *UGT2A1/UGT2A2* locus are associated with increased risk of COVID-19–related acute loss of smell, with the gene product being expressed in SUS cells, consistent with the primary site of infection (41, 45). Functional impairment of these genes contributes to loss of smell (41).

## Factors contributing to OD

### Virus strain

The type of virus strain likely contributes to the variation in the prevalence of OD. During the first pandemic waves, meta-analysis studies showed that the D614G virus was the dominant strain among cases with a high prevalence of OD (46). The incidence of OD after Omicron infection was relatively low (10, 47). The global prevalence of OD in adults was reduced to 3.7% during the Omicron wave (40).

Omicron has reduced effects on olfaction compared to previous VOCs. One reason for this is that support cells are the prime targets of previous VOCs for host cell entry *via* cell surface membrane fusion facilitated by TMPRSS2 (48). A previous study observed the replication of the ancestral strain D614G, the Delta strain, and the Omicron strain in the olfactory mucosa of hamsters, and found that the replication of D614G was the most prominent (49). D614G appeared to be more infectious toward the support cells in the OE (46, 50). The support cells in the OE abundantly express TMPRSS2 (51). The Omicron variant’s lower intracellular S1/S2 cleavage efficiency results in reduced S2’ processing (52), indicating that it is less fusogenic (52, 53); a more detailed description is provided in the section titled “Factors contributing to OD.” This confirms that the Omicron spike is less efficient to enter cells through the TMPRSS2-dependent entry pathway and appears to favor the endocytic pathway (52, 54, 55).

Compared to D614G, Omicron has less efficient replication in the olfactory mucosa (49). The support cells in the OE may be less infected by the Omicron variant (51), resulting in a reduced occurrence of OD. Omicron may spare the olfactory epithelium due to its lower solubility in mucus (51); although most pathogens dissolve in and penetrate the mucus layer (56), how SARS-CoV-2 penetrates the mucus to infect the nasal epithelium remains unclear (57).

The Omicron variant significantly replicated in the human upper airway bronchi (54). Omicron replicated in A549-ACE2 cells but not in A549 cells, indicating that Omicron uses ACE2 as the receptor for infection (53). Extensive ACE2 expression in human bronchi may explain the enhanced replication of SARS-CoV-2 in the bronchi (54). In addition, the S protein of Omicron is sensitive to low-pH-induced conformational changes and adapts to use the low-pH endosomal entry route (52). The lower-pH environment in the upper airway seems more suitable for Omicron survival. Co-expression of ACE2 and cathepsins was more abundant compared to co-expression of ACE2 and TMPRSS2 in the upper airway, which may explain the increased replication of Omicron in the bronchi (54). Moreover, Omicron utilizes the endocytic pathway and has the potential to broadly infect cells with ACE2 expression, regardless of the presence of TMPRSS2. This suggests a ubiquitous ability to expand the cellular infection spectrum (54).

Another reason behind Omicron’s reduced impact on olfaction is that the Omicron variant dramatically spreads *via* the ciliary transport/microvilli reprogramming pathway (57). In general, cilia are classified as motile cilia and nonmotile (or primary) cilia. They are involved in controlling cell fate in the airways (58). Motile cilia beat regularly and serve a mechanical function. Nonmotile cilia serve as sensory organelles in olfactory cells (59). Motor cilia are essential for mucociliary clearance in differentiated epithelial cells (58).

SARS-CoV-2 traverses the mucus layer using motile cilia as tracks *via* the ACE2 receptor to access host cells; mucociliary transport assists in the spread of viruses (57). In this process, there are two models. In model 1, at the cilium surface, SARS-CoV-2 binds to ACE2, and TMPRSS2 activates S protein for ciliary membrane fusion. In model 2, an SARS-CoV-2 –ACE2 complex is transported from the tip of the cilia to the cell body, and the virus fuses with the cell membrane *via* an endocytic-independent process (57). Notably, these models are only applicable to respiratory epithelial cells with motile cilia, and they do not apply to olfactory neurons that have non-motile cilia. The Omicron variant increased the affinity for binding to the ACE2 receptor (60) and accelerated viral entry by binding with higher affinity to motile cilia (57). However, the microvilli did not express ACE2 and TMPRSS2 and did not participate in the SARS-CoV-2 viral entry. SARS-CoV-2 facilitates viral egress by promoting the formation of highly extended and branched microvilli (57).

Currently, the relationship between different virus strains and the occurrence of OD has begun to emerge, and comparison between Omicron and previous VOCs showed that the SUS cell type and TMPRSS2 played important roles during the first pandemic waves and was associated with OD. The mechanism of action needs to be explored further, which will help us better understand the impact of the virus on the human body. Furthermore, this will help us devise more appropriate countermeasures. We believe that OD is a protection mechanism, with SUS cell death exhibiting protective effects against the invasion of the virus.

### Cell entry mediator

The expression of ACE2 in the nasal epithelium is lowest in young children and increases with age (61). Less severe SARS-CoV-2 infections in young children could be aided by lower ACE2 expression and high innate immune responses (62); the aged population prone to SARS-CoV-2 infection may be associated with higher ACE2 expression and low immunity (63). Some authors have argued that, during previous coronavirus epidemics, older people might have had better immune protection because they were more likely to have been exposed to coronaviruses than younger people. Obviously, this does not apply to COVID-19. Regardless of whether individuals have been exposed to the SARS-CoV-2 virus or not, the progression of the disease depends on the immune mechanisms of both older and younger individuals (64). Aging inevitably leads to a reduction in both the number and function of the immune system, making the elderly more susceptible to infections and less capable of responding effectively to pathogenic challenges (65, 66). Children have stronger innate antiviral responses when infected with SARS-CoV-2 than adults (67). Similarly, highly efficient innate immune responses in the nose could be a reasonable explanation for less severe SARS-CoV-2 infection. Research indicates that there is an inverse correlation between age and immune cell proportion in the nasal mucosa (68).

TMPRSS2, a member of the TTSP family, is considered to play a role in epithelial cell biology (69). *Tmprss2* is an androgen-responsive gene (70, 71). TMPRSS2 genes are highly induced by androgens acting through the androgen receptor (72–74). TMPRSS2 is expressed on the surface of epithelial cells of the upper and lower respiratory systems (75), and its role in respiratory virus infections, such as SARS coronaviruses and influenza viruses, is well established (76).

In COVID-19, TMPRSS2 plays a vital role in priming SARS-CoV-2 spike protein (S protein) binding to the ACE2 receptor for viral entry. The S protein undergoes a proteolytic cleavage between the S1 and S2 subunits, allowing viral and cell membrane fusion (77, 78). The S1 subunit contains the receptor-binding domain (RBD) and is responsible for receptor binding, while the S2 subunit is responsible for cell membrane fusion (79). In non-neuronal olfactory epithelial cells, TMPRSS2 expression appears much higher than in olfactory receptor neurons (48, 80). With more TMPRSS2-mediated infections, the probability of OD occurrence increases. One study provided new insight into the role of TMPRSS2, in that the polymorphism in the TMPRSS2 gene is associated with the disease severity of COVID-19 (81).

However, the support cells in the OE express a high level of TMPRSS2, and these target cells in the OE may be less often infected by the Omicron variant (51). This could contribute to reduced OD prevalence during the Omicron wave. A meta-analysis revealed that the host factor is most likely a glycosyltransferase, a type of enzyme that plays a dominant role in the association of OD with SARS-CoV-2 (40). This meta-analysis also revealed that the ethnic profile of Omicron-induced OD prevalence was positively associated with the UGT2A1 risk allele frequency (40). One implication of these new changes is that Omicron variants are more likely to be metabolically responsive, at least in the nasal mucosa. Because glycosyltransferases are metabolic enzymes involved in biotransformation, metabolic reactions mediated by glycosyltransferases may have been affected.

Transmembrane protease serine 4 (TMPRSS4), a new cell entry mediator of SARS-CoV-2 infection, exhibits high expression in regions that are connected with the sense of smell and taste (82). TMPRSS2 and TMPRSS4 are two mucosa-specific serine proteases that promote SARS-CoV-2 virus entry into host cells (83). TMPRSS2 and TMPRSS4 work together to accelerate S protein cleavage, leading to increased membrane fusion (83, 84). TMPRSS4 can trigger S-mediated cell–cell fusion, albeit with lower potency than TMPRSS2 (83). Similar to TMPRSS2, TMPRSS4 increases SARS-CoV-2 viral infection, at least in gut epithelial cells (83). Silencing the expression of TMPRSS4 resulted in a 4-fold decrease in VSV-SARS-CoV-2 replication in human enteroids, which was more significant than that observed in TMPRSS2 knockout cells (83). TMPRSS4 may be involved in modulating immune status and angiogenesis through the nuclear factor (NF)-κB pathway (85). The silencing of TMPRSS4 prevented the activation of the NF-κB signaling pathway (86). The NF-κB pathway is often targeted by viral pathogens to enhance viral replication, host cell survival, and host immune evasion (87). TMPRSS4-overexpressing cells had a higher level of NF-κB (88). The high expression of TMPRSS4 protein in the liver suggests its involvement in the infectivity of SARS-CoV-2 in the liver. This partly explains why immunocompromised patients with liver cirrhosis or hepatic cancer are more susceptible to COVID-19 infection (89). Both ACE2 and TMPRSS4 expression levels were found to be upregulated in epithelial brushing and bronchial biopsy samples of patients with COPD (89, 90). Immune responses are more likely to be activated in regions where the cell entry mediator TMPRSS4 is expressed.

In addition, neuropilin-1 receptor (NRP1) facilitates SARS-CoV-2 cell entry and infectivity (91, 92). NRP1, which is expressed by regulatory T cells, is highly conserved in different species (93), is involved in immune regulation, and seems to be immunosuppressive. NRP1 is abundantly expressed in the RE and the OE (91).

### Odorant receptors

The process of smelling begins with odorants binding to odorant receptors (ORs) on OSNs located on the upper surface of the OE (94). Zazhytska et al. monitored changes in infected golden hamsters over a period of 10 days post-infection (dpi) *via* scRNA-seq (95). SARS-CoV-2 infection causes downregulation of ORs and the OR signaling pathway, as well as the reduction of interchromosomal OR contacts in both humans and hamsters (95). The widespread downregulation of OR genes is the most striking transcriptional change observed in infected OE (95). A similar phenomenon was observed in another study, in which OR genes were shown to be downregulated in the OE of SARS-CoV-2-infected animals (94).

In hamster, significant OR downregulation was first observed at 2 dpi, which then peaked at 4 dpi and was maintained at 10 dpi, while a decrease in SUS cells was observed at 1 dpi that was amplified at 3 dpi (95). OR downregulation was delayed compared to the decrease in SUS cells. The timing of OR downregulation was found to be too late for triggering OD (19). The SUS cell damage can physically separate the SUS cells from the olfactory sensory neuron (OSN) within 2–3 h (19). Methimazole is an olfactory toxicant, and methimazole-induced damage of the olfactory mucosa (OM) of mice was analyzed using transmission electron microscopy; after 2 hours of methimazole administration (50 mg/kg), the degeneration of SUS cells was observed, but minimal damage to the neurons, except for loss of cilia (96). This indicated that in some pathological cases, neuronal cilia were rapidly susceptible to loss of SUS cells. OD is evident as early as 2 days after injury of SUS cells in most animal models (19). Injured SUS cells most likely trigger OD through loss or dysfunction of cilia. OR downregulation may contribute to the duration of OD (19).

OSNs detect volatile odors through ORs that are localized on their neuronal cilia within the nasal airspace (45, 97). OSNs have approximately 10–20 cilia that project from their single dendrite and extend into a thin layer of mucus (~30 µm) that covers the OE surface in the nasal cavity (98). Virus infection can cause downregulation of ORs, and the glucose-trafficking mechanisms may play an important role in this process. The SUS cells uptake glucose from the blood vessels through glucose transporter 1 (GLUT1) and secrete it into the mucus through glucose transporter 3 (GLUT3). Within the mucus, olfactory cilia utilize their GLUT3 to uptake glucose and generate ATP *via* glycolysis, despite their lack of mitochondria (19). After the death of support cells, the energy required to maintain cilia is no longer supplied by the SUS, causing the cilia to retract. This retraction prevents ORs from being trafficked to the cilia, ultimately causing a halt in their production (19). The removal of glucose and the inhibition of glycolysis or oxidative phosphorylation have been shown to impair the odor response (99). Additionally, the downregulation of ORs and other signaling molecules is mediated by the immune system (19, 100). Sterile innate immune activation leads to a reduction in OR expression across the OSNs in OE (100). Research shows that chronic expression of IFN-γ in the mouse OE leads to significant lymphocytic inflammation, which results in a notable decrease in electrophysiological responses to odorants (101). A recent study has also shown that the induction of antiviral type I interferon signaling in the mouse OE was linked to reduced odor discrimination and decreased RNA levels of ORs (100). Moreover, SARS-CoV-2 infection leads to a considerable interferon response and a marked decrease in OR activity in the OE (101).

Reduced OR transcription is one of the few distinctive features of an infected OE (95). Transcriptomic and quantitative proteomic analyses of the OE samples of SARS-CoV-2–infected mice showed that 929 genes and 507 proteins underwent regulation. Gene enrichment analyses showed a strong antiviral IFN response in the OE (94). KEGG pathway enrichment analysis showed that “olfactory transduction” genes were significantly enriched in the downregulated transcripts and proteins in the OE, and the dramatic OR decrease was simultaneous with a significant interferon response (94); therefore, determining whether there is a connection between them is worth investigating. Additionally, in both the human and hamster OE, the widespread, persistent, and strong downregulation of OR genes, as well as the downregulation of other key genes for odor perception, offers a possible explanation for COVID-19-induced OD (95).

Genes of the major histocompatibility complex (MHC) (human leukocyte activation, HLA) are in close physical linkage to ORs (102). A study identified 36 human MHC-linked OR loci (103). Recent studies have suggested that the olfactory and immune systems interact, and that HLA molecules may be a bridge connecting olfactory and immune systems (102). OD can be prolonged by the immune responses, including alterations in the proinflammatory cytokines IL-6, TNF-α, NF-κB, and IFN-γ (101, 104–108). Both the knockout of TNF-α receptor (TNFR)-1 gene and that of TNFR-2 gene in mice demonstrated decreased inflammation in the OE (102). OD may be associated with autoimmune state (109). In addition to changes in ORs, enzymes that are expressed in the olfactory epithelium, such as UDP glycosyltransferase, eliminate the odorants that bind to ORs when they enter the nasal cavity (40, 41).

Both cAMP and cGMP are present in human nasal mucus, and their level is lower in patients with OD than in normal subjects, suggesting their connection to olfactory pathology (110). Intracellular cAMP levels increase, while the production of proinflammatory mediators decreases and the production of anti-inflammatory mediators increases (111). ORF7b is likely involved in the dysfunction of ORs (112). The occurrence of OD in COVID-19 patients is not the result of a single factor, but a host of factors.

### Age and gender

The age, gender, and immune state of the host are considered to be prominent biological factors that determine the elimination of pathogens that cause infectious diseases (113). Symptoms of OD and GD were more common in females and in younger patients (114, 115).

Gender and age can be considered as intrinsic factors of susceptibility to viral infection. Patient sex impacts innate and acquired immune responses. Animal and clinical studies have shown that the innate immune cells in females, including monocytes, macrophages, and dendritic cells, are generally higher in number and activity relative to males (113). Females have a reduced susceptibility to viral infections because they often elicit stronger immune responses than males (116). Stronger immune responses might be an underlying reason for the increased development of symptoms of infection among females compared to males (116). Females seem to be more susceptible to OD and GD. In the study cohort of Lv et al., 64.1% of patients with OD were female (117). The human X chromosome contains many genes associated with immune responses, including the innate and adaptive immunity genes, which will affect antiviral immune response.

## Roles of the cytokine IL-6

IL-6 is an inflammatory cytokine overproduced in a spectrum of clinical conditions, including renal disease, cardiovascular disease, and hepatitis (118). IL-6 induces acute-phase proteins, including C-reactive protein, fibrinogen, serum amyloid A, haptoglobin, complement components, and α1-antichymotrypsin (104, 118), and activates coagulation cascades, possibly resulting in disseminated intravascular coagulation (104). IL-6 can be produced by immune cells like macrophages, dendritic cells, T lymphocytes, and B lymphocytes (119, 120), and IL-6 often functions in association with the innate immune sensing system to link innate and adaptive immunity and to control antibiotic effects (119).

However, the role of IL-6 in OD is controversial, with only some of the studies reporting a correlation between IL-6 levels and OD. Levels of IL-6 in the nasal mucus, saliva, and plasma of hyposmia patients were significantly higher than in controls (118). In hyposmia patients, mean levels of IL-6 in nasal mucus were 2.6 times those in controls (118). A higher concentration of IL-6 in the nasal mucus was considered to play a role in biochemical pathological processes underlying OD (118). OD patients in COVID-19 had higher levels of IL-6 (104). When the higher levels of IL-6 returned to normal (1–7 pg/mL), patients began to recover from OD (104). Groups with OD caused by Omicron infection had higher IL-6 levels (121).

More recent studies reported no such correlation between IL-6 levels and OD. Using psychophysical olfactory scores, Vaira et al. found that the correlation between IL6 levels and OD was weak and not significant (122). Regarding this result, Won Sriwijitalai et al. commented that IL-6 is a continuous parameter, but the psychophysical olfactory scores are obtained from a scoring system and represent a numerical scale from a basic counting; therefore, the correlation is invalid (123). Additionally, the direct proportional correlation between IL6 levels and OD conflicts with what has been reported in COVID-19 patients with OD. Although the serum IL-6 levels of all COVID-19 patients increased compared to normal serum IL-6 levels (7 pg/mL), patients with OD had significantly lower IL-6 levels (16.72 ± 14.28 pg/mL) than patients without OD (60.95 ± 89.33 pg/mL) (p = 0.026) (124). Another study of 218 COVID-19 patients showed that the subjects with OD had slightly lower serum IL-6 levels than those without OD (3.71 *vs*. 6.11, *p* < 0.001) (47). Additionally, the above study found no significant correlation between IL-6 and the occurrence, severity, or recovery of OD after Omicron infection (47). A meta-analysis showed that COVID-19 patients with chemosensory disturbances had lower levels of IL-6 than patients without chemosensory disturbances (125); other meta-analyses of studies that examined systemic IL-6 levels are consistent with this result (124, 126). Therefore, how IL-6 functions in nasal mucosal immune responses and how it affects the immune system of the body remains to be clarified.

A previous study reported that IL-6 levels were significantly higher in severe COVID-19 cases than in non-severe cases (25.2 *vs*. 13.3 pg/mL; p < 0.001) (127). IL-6 showed increased expression along with disease severity (128), and it appears as a potential predictor for the progression of COVID-19 to severe disease. A meta-analysis reported an IL-6 cut-off value of 55 pg/mL for severe disease course (129). A significant positive correlation was observed between IL-6 and mild disease groups (130). Lower IL-6 levels were related to mild disease course in COVID-19 (124, 131).

In a 2020 sub-cohort study of 278 COVID-19 patients in China and Germany, the incidence of OD or GD in mildly, moderately, and severely ill patients was 53 of 98 (54%), 40 of 107 (37%), and 11 of 63 (17%), respectively (4). A study of 417 COVID-19 cases with mild and moderate disease courses showed that OD was present in 85.6% of cases (124), supporting observations that COVID-19 patients with OD have a milder disease course as demonstrated by clinical, laboratory, and imaging findings (124). COVID-19 patients without OD had a significantly higher percentage of parenchymal involvement (unilateral/bilateral involvement, lower/upper lobe predominance, diffuse/peripheral/central/mixed involvement) compared to patients with OD (p = 0.006; p < 0.01) (124). On control chest CT, COVID-19 patients with OD had significantly lower rates of progression (p = 0.016; p < 0.05) compared to those without OD (124). The prevalence of OD in severe patients was lower than that in mild-to-moderate COVID-19 cases (132).Although there has been a decline in the occurrence of OD, it is still present during the Omicron wave. This shows that the entry mechanisms play the dominant role in the reduction of OD occurrence.

## Infection of SUS cells

SUS cells in the OE play key roles in odor sensing and supporting olfactory neuron metabolism (35, 43). SUS cells are usually involved in odor processing *via* endocytosis of the odorant-binding protein complex, detoxification of enzymes belonging to the cytochrome P450 family, maintenance of cilia of mature olfactory receptor neurons, and preservation of epithelial integrity and local ion balance (37, 43, 99, 133, 134). In the SARS-CoV-2-infected OE of golden hamsters at 1 dpi, 47% of the total infected cells were SUS cells, and SUS cell reduction was associated with an increase in microglia and other immune cells (95). SUS cells are the major target cell type in the olfactory mucosa (35, 48, 50). SUS cells express high levels of ACE2 and TMPRSS2 (32, 34, 37). Macrophages play a major role in innate and adaptive immunity. They are present within all tissues of the body and can be involved in injury inducement or repair promotion (135).

Macrophages extend pseudopodia when contacted by invading pathogens (136) and play a key role in cell-mediated neuroprotection (136). The nucleocapsid protein (NP) was observed to colocalize with CD68+ macrophages, suggesting that the immune cells were direct targets of SARS-CoV-2 (137). Another study observed NP co-immunostaining with the inflammasome NLRP3 and CD68+ macrophages in the brain tissue and postmortem lung (138). Mucosal infiltration by CD68+ macrophages may contribute to COVID-19-related OD (135). This is a self-protection mechanism; macrophages eliminate pathogens by phagocytosis.

Furthermore, SUS cells receive and eliminate viruses; in the process, SUS cells die, but they can regenerate. Supporting cells in the olfactory epithelium have the ability of phagocytosis (139–143). The role of the phagocytosis of SUS cells in COVID-19 infection needs to be explored. SUS cells protect olfactory neurons from being damaged by hazardous molecules (144). In COVID-19, SUS cell death may provide protective effects against the invasion of the virus. Furthermore, cell death and regeneration occur much faster in SUS cells than in olfactory neurons, and thus the rapid replenishment of SUS cells should be equivalent to the rate of rapid recovery from OD in the majority of COVID-19 cases during the first pandemic waves (133).

Two mechanisms underlying OD occurrence in COVID-19 have been proposed: the infection of SUS cells and the inflammatory reaction of the nasal epithelium. The former triggers OD, the latter likely prolongs OD. These two alternative mechanisms act in parallel; the infection of SUS cells is more important for OD because SUS cells are more likely to be the entry point of SARS-CoV-2 than olfactory neurons. Furthermore, SUS cells abundantly express TMPRSS2 and play a major role in the olfactory epithelium. OD occurrence in COVID-19 has revealed crucial roles of SUS cells.

## Inflammatory reaction of the nasal epithelium

Inflammation of the olfactory epithelium is significant. It likely prolongs OD because it can be detrimental to normally occurring regeneration of the neuroepithelium and therefore delays prompt recovery of the sense of smell. The epithelium, one of the front-line protections against viral infections, secretes cytokines and recruits immune cells to the site of inflammation (145), which induces injury to the OE. Local cytokine-mediated inflammation contributes to OD (101). During SARS-CoV-2 infection, elevated levels of cytokines and antiviral genes such as IFN-γ were detected in most of the infected samples, indicating active antiviral gene expression (95). The IFN-induced transmembrane (IFITM) proteins, a group of interferon-stimulated downstream genes encoding several antiviral innate immune effectors, play an important role as host self-restriction factors and exert antiviral activity against various enveloped viruses by blocking the fusion between viral and cellular membranes (146). IFITM proteins have immunological and clinical significance in innate immunity and differ from many classical interferon effectors in that their distinctive capability is that they impact virus entry processes rather than intracellular virus replication (147, 148). IFITM3 interacts with vesicle membrane protein–associated protein A (VAPA) and hinders its association with oxysterol-binding protein (OSBP), resulting in an increase in endosomal cholesterol content and thus obstructing viral entry (149, 150).

A study has found that each of the human IFITM proteins—IFITM1, IFITM2, and IFITM3—can inhibit SARS-CoV-2 infection (148). IFITM proteins can also limit the entry of SARS-CoV into lysosomes that depend on cathepsin, but they are incapable of restricting trypsin-induced fusion happening at or near the plasma membrane (151). Notably, TMPRSS2 is a trypsin-like protease (152) that exerts its effect on viral entry. Studies suggest that human coronaviruses can evade IFITM protein restrictions at high levels of TMPRSS2, which is consistent with this observation, and overexpression of TMPRSS2 reduces the inhibitory effect of IFITM3 on SARS-CoV-2 infection (148, 153, 154). One study has revealed that the degradation of IFITM2 and IFITM3 proteins is functionally associated with the enhancement of SARS-CoV-2 infection (152); the activation of TFEB, which is a master regulator of lysosome function regulated by mTOR, triggered the turnover of IFITM proteins and the enhancement of SARS-CoV-2 infection (152). At present, little is known about how SARS-CoV-2 infection overcomes IFITM2 restrictions. A comprehensive exploration of the effects of IFITM proteins on SUS cells could enhance our understanding of OD.

Acute injury of the olfactory neuroepithelium stimulates a local inflammatory response and cytokine production (107), unlike the chronic inflammatory state, the acute inflammatory response promotes regeneration of the olfactory neuroepithelium (107). NF-κB signaling is involved in the acute inflammatory response and neuroregeneration process in the OE (107). Initiation of neural stem cell differentiation *in vitro* requires NF-κB signaling (107, 155). Chronic expression of IFN- γ in the OE initiates a local inflammation and attenuated olfactory function without concomitant neuroepithelial damage (101). IL-6 acts *via* TNF-α or neuropoietin (NP)-activating apoptotic pathways to disrupt olfactory function (104). In turn, IL-6 levels normalize when OD and GD resolve (104). TNF-α is the most relevant to OD amongst the cytokines, and a notable expansion of the olfactory submucosa is a result of chronic local TNF-α expression (106). TNF-α induces apoptosis of olfactory neurons in OE explants (156). Surprisingly, SUS cells were the source of TNF-α (106). A normal histologic appearance and functional recovery of the neuroepithelium were observed when TNF-α expression was discontinued (106). Knockout of TNF-α receptor (TNFR)-1 gene in mice demonstrated decreased inflammation in the OE (108), and similar results were found for the knockout of TNFR-2 gene (102, 105).

Brann et al. proposed that inflammation blocking effective odor conduction, altering the function of OSNs, deteriorating signaling, or causing diffuse architectural damage of the OE may be the mechanisms for OD (32). Innate immune cells like neutrophils, monocytes, and macrophages could also produce the desquamation of OE through inflammation (157–159). Bourgon et al. proposed that immune responses make the OE destruction (and OD) worse, suggesting that innate immune cells play a major role in the destruction of OE (158). Interestingly, the impairment of neutrophil activity resulted in a reduction of SARS-CoV-2 infection levels in the OE of infected hamsters (158). This finding suggests that neutrophils play a counterproductive role, resulting in the release of infected cells into the nasal cavity lumen and thereby enhancing viral spread during the early phase of SARS-CoV-2 infection (158). The authors observed a strong cleaved caspase 3 signal that partially colocalized with desquamated cells in the nasal cavity lumen, showing a 5- and 14-fold increase at 1 and 2 days post-infection, respectively, compared to the OE at the early stages of infection (158). OSNs, but not SUS cells, undergo Caspase 3 apoptosis once released in the nasal cavity (158). The inflammatory response of the olfactory neuroepithelium could affect the function of olfactory neurons, resulting in prolonged duration of OD (19). To a certain extent, immune reactions in the OB and other CNS regions cause OD (33). An aggressive immune response can deteriorate neuronal cells and stem cells (160).

A multi-tissue study of immune gene expression profiling has highlighted the importance of the nasal epithelium in COVID-19 severity (161). Strong cleaved caspase-3 signals implying cell apoptosis have been observed both in infected and noninfected cells in the olfactory mucosa of OD patients in COVID-19 (162). The observed OD in COVID-19 patients is likely linked to a rapid and significant desquamation of the OE due to infection of SUS cells with SARS-CoV-2 (163). This leads to the recruitment of immune cells in both the OE and the lamina propria (163). For infected hamsters, as apoptosis does not occur significantly in the OE during the initial phase of SARS-CoV-2 infection, the desquamation of the infected OE may be related to the infiltration of immune cells (158). One study suggested that neutrophils are recruited as early as 1 day post-infection and that their recruitment persists at 2 days post-infection alongside the arrival of Iba1+ and CD68+ cells (158). Iba1+ cells are significantly more prevalent in the infected OE compared to CD68+ macrophages and MPO+ neutrophil cells, with the latter two being predominantly present in the desquamated cells filling the lumen of the nasal cavity in SARS-CoV-2–infected individuals (158). There is a significant correlation between OE damage and the presence of Iba1+ cells (macrophages/microglia), CD68+ cells (activated bone-marrow-derived macrophages), and MPO+ cells (neutrophils) (158).

## Discussion and future direction

COVID-19 is an immune-mediated disease that can affect multiple organs (164), so the local area cannot be separated from the systemic context, and we believe a major contributing factor in OD is immunity. SARS-CoV-2 infection alters the gene expression profile in the nasal turbinate tissues, leading to upregulation of 309 and downregulation of 62 genes. The most upregulated genes are antiviral genes (165). Ingenuity Pathway Analysis (IPA) showed that SARS-CoV-2 infection primarily induced antiviral and proinflammatory pathways related to innate immunity, interferon signaling, and immune cell activation in the nasal mucosa (165). Host immune responses may contribute to the downregulation of genes involved in olfactory signal transduction (19). Children have a higher basal expression of melanoma differentiation-associated protein 5 (MDA5) and retinoic acid-inducible gene (RIG)-1 on nasal epithelial cells, dendritic cells, and macrophages. Thus, they have a stronger innate immune response to SARS-CoV-2 (166). In both children and adults, there is an IFN response to infection with SARS-CoV-2 in the nasal mucosa (167).

In addition to the factors and mechanisms contributing to OD mentioned above, it is worth noting that the S protein of SARS-CoV-2 is more exposed due to lower glycosylation density compared with SARS-CoV and MERS-CoV, and it is more effective in eliciting a humoral immunity response (168). The virus may trigger different immune responses, and different symptoms can occur during this process.

According to Le Bon et al. (169) and Hopkins et al. (170), OD may serve as a mechanism and survival strategy that the body uses to protects itself. This is supported by chest CT findings showing the distribution of parenchymal involvement, which confirmed that all OD cases had less than 25% parenchymal involvement (124). Chest CT findings showed a lower rate of progression and more rapid radiological recovery in OD cases (124). A previous study indicated that, during the Omicron wave, the clinical course of the disease was milder for the Omicron variant than for non-Omicron variants (171). The Omicron variant of COVID-19 primarily infects the upper respiratory tract and shows decreased infectivity in the lungs. Furthermore, the disease associated with this variant was found to exhibit a less severe clinical course (172, 173). Despite the reduction of OD with Omicron, patients infected with Omicron usually have a much milder disease of COVID-19, which seems to contradict the notion that OD is associated with (and may contribute to) a milder disease course. This contradiction illustrates that the progression of a disease is influenced by multiple factors, including external factors (such as invasion by different strains) and internal factors (such as an individual’s immune response to the strain).

Minimal inflammatory infiltrates have been reported in nasal biopsy specimens, indicating that local inflammation is not the sole contributory factor to OD during COVID-19 infection (135). The presentation of OD in SARS-CoV-2 infection without concomitant nasal inflammatory symptoms is another indication of this possibility (34).

Furthermore, one meta-analysis reported the mean duration of OD was less than 9 days for pre-Omicron infections (174). Despite the damage, we have the ability to repair ourselves and recover from OD. SARS-CoV-2 seems to induce OD, but not permanent injury in most cases. A substantial proportion of patients did not fully recover from OD and might develop long-lasting changes in their sense of smell (175–177). T cell-mediated inflammation persists in the OE suggesting a mechanism for long-term post-COVID-19 smell loss (45). Exploring the recovery mechanism is essential for the development of improved medical treatments for COVID-19–related OD. Patients should be prepared for the probability that OD may persist for years, and providers should guide them to cope with psychosocial obstacles (178). Stem cell therapy has been proposed for COVID-19–associated persistent OD (179).

The molecular mechanism underlying OD is rarely described in detail. The exact mechanisms of OD remain unclear (48), so further research on the host–virus interaction in human nasal cells is needed. If the exploration of mechanisms can be integrated with the proper understanding of clinical symptoms, then the most appropriate treatment for these patients may be forthcoming.

## Author contributions

SC conducted literature research, organized, and wrote the manuscript. SW summarized relevant content, proofread, and revised the review. All authors listed have made a substantial, direct, and intellectual contribution to the work and approved it for publication.
